# Reinventing conventional participation: A model to mitigate political disaffection in Lambayeque, Peru

**DOI:** 10.12688/f1000research.162192.1

**Published:** 2025-03-17

**Authors:** Gustavo Adolfo Ventura Seclén, Juan Amilcar Villanueva Calderón, Ida Blanca Pacheco Gonzales, Rocio Desiret Yarlaque Muñoz, Germán Auris Evangelista, Segundo Roberto Vásquez Bravo, Pilar Mercedes Cayllahua Dioses, José Santos Ventura Sandoval, Renato de Jesús Granados Rodríguez

**Affiliations:** 1 Trujillo, Universidad Cesar Vallejo, Trujillo, La Libertad, Peru; 2 Universidad Nacional Toribio Rodriguez de Mendoza de Amazonas, Chachapoyas, Amazonas, Peru; 3 Universidad Nacional Pedro Ruiz Gallo, Lambayeque, Lambayeque, Peru

**Keywords:** conventional participation, political disaffection, trust, perception actitud

## Abstract

**Background:**

In a district of the Lambayeque province, considerable levels of political disaffection are evident, manifesting not only in the alienation from power but also in a significant gap between citizens and government institutions. Although efforts have been made to continue the participatory trend in democratic institutions, innovative proposals for social intervention in political processes and decision-making are still needed. The study aimed to propose a model of conventional participation to mitigate political disaffection in Lambayeque, Peru.

**Methods:**

The research was quantitative, with a non-experimental, descriptive-propositional design. It employed a survey technique applied to a randomly selected sample of 506 citizens from a total universe of 19,342 voters.

**Results:**

The results indicate that the implementation of a conventional participatory practice, tailored to the sociocultural peculiarities of the district, positively impacts the political perception of the population.

The findings reveal that a large portion of the Lambayeque population has disconnected from politics in everyday life, registering a general malaise that may reflect in future elections, as interest that does not translate into action is meaningless.

**Conclusions:**

General distrust in institutions, particularly the sentiment of corruption, has fostered this detachment; moreover, the results reflect a rejection of the political system. Despite many being informed about politics, the feeling of participation remains impotent.

## Introducción

Traditional forms of participation, such as popular consultations, public hearings, and municipal councils, have proven to be unviable in actively involving citizens in both local and national politics ( Berlanga et al., 2023). This is specifically evident in Lambayeque, where geographical, socioeconomic, and technological barriers hinder access to these spaces for social engagement ( Díaz, 2022). The existing methods of citizen participation are not adapted to the realities of the population, leading to a disaffection from politics, where citizens negatively assess their authorities and distance themselves from political matters ( Duárez, 2022). In this regard, it is necessary to create a renewed model that leverages new forms of participation and high-tech tools to increase society’s interaction with authorities ( Jara et al., 2021).

On the other hand, the inequalities between urban and rural areas still create a deep gap in accessing the political participation process. Rural communities, with their strong ties to the land and agriculture, often find themselves disconnected from political decision-making spaces, sinking into a sense of anti-political alienation ( Canaza, 2024). This phenomenon not only limits effective representation but also hinders the design of state policies that adequately respond to the specific needs of these communities. Therefore, in designing a new model of conventional participation through a more open and equitable system, social integration must be fulfilled ( Martínez et al., 2022).

In this context, a fundamental point in remodeling the participation model is the fusion of digital technologies, which offer a unique opportunity to overcome the physical and social barriers inherent in conventional participation. However, bringing technological tools to rural areas poses significant challenges, such as limited internet access and digital literacy ( Jara et al., 2021).

To some extent, the proposal to reinvent the participation model involves not only introducing new technologies but also broadening the approach to civic education ( Rodríguez, 2024). In this sense, the politics of disaffection is closely related to the lack of knowledge about how participation operates and exercising citizens’ rights. Therefore, it is essential to implement programs that strengthen not only political culture but also citizens’ civic sense ( Moyano & Solís, 2021). These programs should lead citizens not only to understand the importance of participation but also to provide them with concrete tools to interact effectively in both politics and society ( Megías & Moreno, 2022).

Civic education should be part of the participatory process as a prior and ongoing step, thus ensuring informed and committed participation. Furthermore, equal opportunities must be guaranteed for all social classes, regardless of geographical location, educational level, or virtual access ( Freire, 2023).

Methodologically, this study takes a quantitative approach with a non-experimental and descriptive proposal, allowing for the examination of various factors that increase the level of political disaffection among citizens.

Political disaffection in Lambayeque requires a comprehensive response that includes not only changes in traditional participation mechanisms but also accessible technological tools and civic education programs that encourage citizens to engage with subnational politics. The reconstruction of the conventional participation model needs not only to be digitized but also to foster an inclusive mindset regarding participation, thus instilling greater credibility in political institutions ( González & Salvatierra, 2021).

The study aimed to propose a model of conventional participation to mitigate political disaffection in Lambayeque, Peru. The specific objective was to determine the factors that lead to political disengagement in Lambayeque, Peru.

Finally, the following hypothesis was proposed: The implementation of an innovative citizen participation model in Lambayeque, Peru, will contribute to mitigating political disengagement.

## Marco Teórico

Regarding international background, the study by Neves (2024) was cited, which analyzed the democratic discontent of youth and how this behavior can lead to democratic deconsolidation in various European contexts. The results revealed that the discontent of young people has become institutionalized non-democratic patterns, which has diminished their commitment to democracy. The study by Megías and Moreno (2022) aimed to verify the impact of social participation on reducing political disaffection in urban municipalities or districts in Germany, France, and Spain, with results indicating that as the level of social participation increases, a defined segment of politics can benefit. Similarly, Cárdenas (2022) identified the different characteristics or peculiarities of the political culture of voters regarding municipal candidates in a city like Bogotá, resulting in the finding that limited political knowledge allowed unsuitable political representatives to be elected, generating a proven disaffection characterized by distrust and distancing from institutions.

On the other hand, Alaminos et al. (2024) in their study propose characterizing political disaffection in a Latin American context, with results indicating that there are alternatives under which it is possible to define a historical-social subject, creating new patterns in democracy.

In the Peruvian context, Núñez Lira et al. (2020) examine the functioning of the structural problems facing politics in Peru, with a special emphasis on how citizens confront representative democracy. The results showed that the level of distrust in political institutions is increasingly higher, leading to a phenomenon of political disaffection over the past few years. Finally, Murakami and Pozsgai (2024) examined the factors that have contributed to the growing crisis of trust and political discouragement in various regions of Peru, concluding that fewer people participate in politics in general, as a consequence of unmet electoral promises.

Regarding the study variables, we have:

### Conventional participation

Regarding the theoretical foundations of the conventional participation program, it aligns with the contribution of participatory democracy by Dahl (1989), which states that in a democratic system, active participation by citizens is fundamental, as it improves policy formulation based on public decisions and the management of social issues.

It also refers to an articulated set of activities or strategies aimed at stimulating the level of citizen participation in formal political procedures, using the means and institutionalized channels that operate within a democratic system ( Castellanos, 2020). This type of measures aims for citizens to engage in traditional participation mechanisms such as voting in elections, joining political parties, attending community meetings, supporting candidates, and public consultation ( Garrido and Sáenz, 2020).

### Political disaffection

Regarding the theoretical foundation of political disengagement, it is based on the theory of the good citizen, which was inextricably linked to being well-informed and predominantly participating in conventional activities such as voting or empathizing with traditional political institutions ( Dalton, 2008). The theory argues that it has undergone a change over time, shifting to become the increasingly individualized modus operandi of citizens who participate mandatorily in traditional political avenues, showing complete disinterest in being part of government rotations ( Rodríguez, 2024).

Conceptually, political disengagement is the process in which individuals and groups detach and interrupt their relationship with political and administrative institutions in a democratic system ( Witteveen et al., 2022). This type of participation is understood as following the formal procedures and channels established by the political-legislative framework of society; in other words, it involves citizen participation through the structures and mechanisms formally established by the government and public institutions, such as elections, public consultations, hearings, citizen committees, and other formal means of civic participation, yet it clashes with the preservation of what is established by the normative framework ( Angarita et al., 2021).

## Methodology

### Type, design, and scope

According to the article, it was an applied inquiry, with a quantitative approach and a non-experimental, descriptive-propositional design ( Hernández & Mendoza, 2018).

### Population and sample

The sample included 506 citizens who are part of the electoral roll of the context under study. The distribution of the sample is indicated according to the following data:

**Table 1.** Characteristics of the sample by sex.

Sex	Process	
	F	%
Male	327	65.4%
Female	179	34.6%
TOTAL	506	100%

*Note:* Instrument (questionnaire) applied to the sample. F is Frequency and % is Percentage.

**Table 2.** Characteristics of the sample by residence area.

Residence	Process	
	F	%
Rural	418	82.6%
Urban	88	17.4%
TOTAL	506	100%

*Note:* Instrument (questionnaire) applied to the sample. F is Frequency and % is Percentage.

### Procedure

This process follows several stages to analyze the chosen study phenomenon within the study context. In the process, there are several important steps: starting with a general idea, formulating the problem, designing the methodology, sampling, collecting and processing the information, and interpreting-discussing the obtained results.

### Techniques and instruments for data collection

It is important to highlight that the technique used in the study was the survey, which was administered through a questionnaire. Regarding political disaffection, 15 questions were taken (with 5 options structured according to the Likert Scale).

### Validity and reliability

The survey instrument was validated by the experienced opinion of individuals with extensive credentials and legitimacy to provide information, evidence, and judgment. The reliability of the tool was assessed using Cronbach’s Alpha coefficient, where the questionnaire achieved a score of 0.720, considered very reliable.

### Data análisis

The data analysis was carried out using IBM SPSS Statistics 26, which facilitated the acquisition of precise and detailed descriptive results for each variable. This approach also enabled the verification of the hypotheses proposed in the research, ensuring the robustness and validity of the obtained results.

### Ethical component

During the research process, 506 selected individuals participated, all of whom had provided their authorization and informed consent, which allowed us to have the necessary data for the development of the study. This consent was obtained in accordance with the principles of intellectual honesty, transparency, respect for intellectual property, and responsibilityThis study has been approved by the Institutional Research Ethics Committee of the César Vallejo University, in accordance with the Code of Research Ethics of the UCV (Code: PP-DG-02.01, Version: 02, Date: 08-29-2024). The approval number assigned to the present study is RCU No. 0659-2024-UCV.

The study was carried out in compliance with the principles established in the Declaration of Helsinki on research in human beings. All participants were informed about the objectives of the study and provided informed consent before participation. The confidentiality of the data, the right to privacy and the possibility of withdrawing from the study at any time without any consequences were guaranteed. For minor participants, informed consent was obtained from their parents or legal representatives, in addition to the consent of the minor, as stipulated in the current Clinical Trial Regulations of the National Institute of Health (INS, Peru).

The study does not involve medical interventions or experimental procedures that put the integrity of the participants at risk. Likewise, the standards of protection and well-being of research subjects will be respected in accordance with institutional guidelines and applicable national and international regulations.

## Results

### Descriptive analysis

Based on the data obtained from the survey applied to the sample, the following data were generated.

**Table 3.** Frequencies and percentages for the variable political disaffection.

Levels	*Political disaffection*	
	F	%
Low Level	185	36.6%
Medium Level	177	35.0%
High Level	144	28.5%
Total	506	100.0%

*Note:* Instrument (questionnaire) applied to the sample. F is Frequency and % is Percentage.

In the group of 506 people that made up this sample, the majority exhibited a Low level of political disaffection, with 36.6%, indicating that they are somewhat disconnected from the political life of their locality. A 28.5% showed high disaffection, suggesting a more distant or indifferent attitude toward politics, while only 35% displayed a medium level of disaffection, indicating that a small group still maintains commitment or interest in politics. The general trend is clearly toward political disaffection, but most subjects are positioned in moderate stances to better address or resolve these issues, leading to their participation being moderately null.

**Table 4.** Level of distrust in institutions.

Levels	*Distrust in institutions*	
	F	%
Perception of Corruption	174	34.4%
Trust in the Judicial System	177	35.0%
Trust in the Political System	155	30.6%
Total	506	100.0

*Note:* Instrument (questionnaire) applied to the sample. F is Frequency and % is Percentage.

In the sample of 506 people, distrust in institutions is reflected as follows: 35% trust in the justice system, indicating a higher degree of distrust regarding this institution. In contrast, 30.6% trust in politics as represented by Congress and the president, which attests to a certain lack of confidence in this area. Finally, 34.4% distrust according to the perception of corruption, which shows a widespread negative perspective on democratic institutions. In summary, trust in the justice system prevails, while distrust is more pronounced in the political system, which is perceived as corrupt.

**Table 5.** Level of Political-Social Distancing.

Levels	*Political-social distancing*	
	F	%
Electoral Participation	178	35.2%
Political Interest	223	44.1%
Electoral Relevance	105	20.8%
Total	506	100.0%

*Note:* Instrument (questionnaire) applied to the sample. F is Frequency and % is Percentage.

The majority of people in the sample show a strong interest in politics, with 44.1% expressing concern, but only 35.2% are committed to actively participating in elections. This suggests that, while many people are drawn to political issues, few actually take action. Additionally, 20.8% believe that election results are not important. Thus, although political interest is high, the low electoral participation highlights a disconnect between enthusiasm for politics and the willingness to engage in electoral decisions.

**Figure 1.**
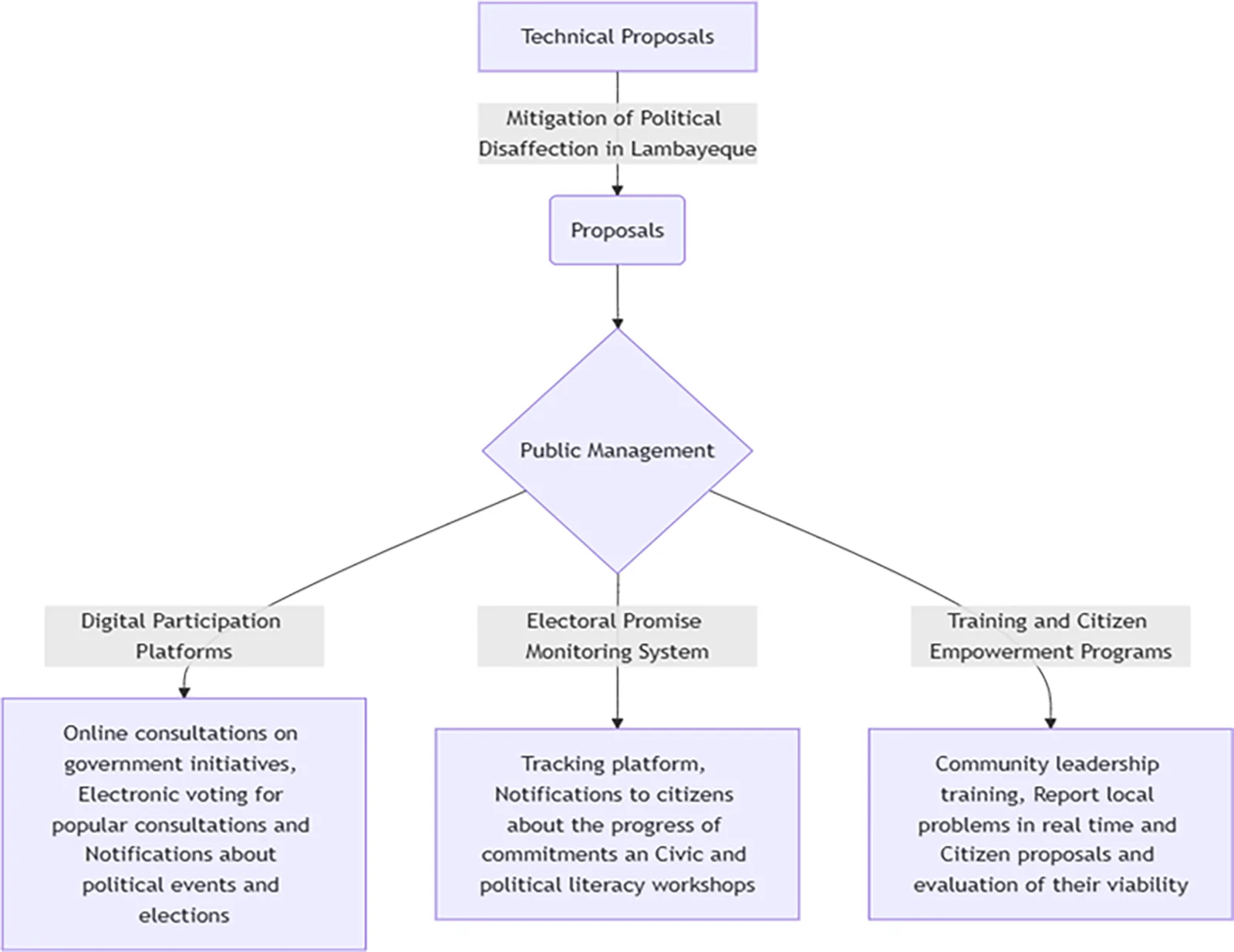
Illustration of technical proposals to mitigate Political Disaffection in Lambayeque. *Note.* Prepared based on the questionnaire data.

The proposals to mitigate political disaffection in Lambayeque aim primarily to return the voice to citizens and create a closer and more transparent relationship with the authorities. These initiatives seek to make politics a more accessible space, where everyone has the opportunity to participate and feel heard.

One of the ideas is the development of digital participation platforms, where this approach leverages technology so that citizens can engage more directly and easily. Through online consultations about government initiatives, anyone could express opinions and contribute ideas on projects that directly affect them. Electronic voting for popular consultations would also facilitate the participatory process, allowing people to vote from the comfort of their homes, without the physical barriers that often limit their participation. Additionally, notifications about political events and elections would keep citizens informed, fostering greater awareness and active participation in political processes.

On the other hand, the system for monitoring electoral promises aims to ensure that politicians are held accountable for what they promise. This system would allow citizens to track the promises made during campaigns and see if they are being fulfilled. The creation of a tracking platform would enable anyone to check, in real-time, whether the promises of their representatives are being realized. An interactive visualization of compliance would help make the information accessible and understandable, while periodic notifications about the progress of those commitments would keep citizens informed and empowered to demand the fulfillment of promises.

Finally, strengthening citizen training and empowerment programs seeks to educate the population and make them more aware of their role in democracy. This includes civic and political literacy workshops, where citizens would learn about their rights, the democratic system, and how to exercise them. Additionally, community leadership training would provide individuals with tools to take active roles in solving local problems, making them key actors in their community. Furthermore, the proposal includes creating mechanisms for citizens to report local issues in real-time, facilitating direct interaction with authorities and ensuring that solutions are quick and effective. Lastly, allowing citizens to propose solutions and evaluate their feasibility would strengthen the sense of belonging and collaboration, giving everyone an active role in improving their environments.

Together, these proposals seek to create a space where politics is seen not as something distant or inaccessible, but as a tool to improve the daily lives of all. By focusing on transparency, participation, and civic education, the goal is to rebuild trust between citizens and their representatives, generating a stronger commitment and a closer relationship between both. With these initiatives, Lambayeque could become a model of citizen participation, where politics is understood as a means to solve common problems, rather than a distant or exclusive process.

## Discussion - Conclusions - Recommendations

The results obtained in this research reflect a clear political disaffection among the population of the Lambayeque district. The majority of respondents show a medium level of political detachment, indicating a disconnection of citizens from the current political situation. However, a significant group also exhibits a high level of disaffection, reflecting a marked disinterest in political processes. This trend aligns with previous studies, such as Neves (2024), which observed high levels of democratic discontent in Europe, especially among young people, which could have parallels in the Peruvian context, albeit with unique characteristics in provincial regions.

The analysis of distrust in institutions reveals that the perception of corruption is one of the factors that most undermines citizens’ trust, affecting 23.3% of respondents. This perception coincides with the findings of Cárdenas (2022), who indicated that both misinformation and distrust in political representatives contribute to political disaffection. Moreover, the judicial system and the presidential administration are viewed with skepticism, with a 44.9% distrust towards the judicial system. These data corroborate the widespread perception that political institutions do not meet citizens’ expectations, something that Alaminos et al. (2024) refer to as key in the disconnection between politics and citizens.

Regarding electoral participation, the results show a low voting rate, indicating that many citizens feel demotivated or skeptical about the real impact of their vote on political decisions. This finding aligns with studies by Murakami and Pozsgai (2024), which detected a similar phenomenon in various regions of Peru, where indifference towards politics seems to have grown, despite interest in political issues. It can be inferred that, in addition to a lack of interest or knowledge, disaffection is also profoundly influenced by the perception of corruption and the ineffectiveness of political and judicial institutions. Studies by Cazorla et al. (2022) on disaffection in Latin America highlight how media can influence the exacerbation or moderation of these feelings of disconnection.

It is important to note that, although political interest remains high, the effectiveness of that interest in actual participation is limited. While citizens are informed about politics, many do not feel that their participation has a tangible impact, a phenomenon also observed in other contexts, as evidenced in Jiménez’s (2022) study on the relationship between social participation and political disaffection. This study indicated that increasing participation does not always reduce the gap between citizens and politics.

In conclusion, the results reveal that a large part of the population in Lambayeque has disconnected from politics in daily life, recording a general discomfort that may be reflected in upcoming elections, as that interest that does not translate into action is meaningless. The general distrust in institutions, especially the feeling of corruption, has fostered this detachment. Moreover, the results indicate a rejection of the political system; however, although many are knowledgeable about politics, the feeling of participation is one of impotence.

## Ethics and consent

In the research process, 506 individuals selected from the population participated. It all began with sending an email to each participant, requesting their informed consent, which we received in writing through the same channel. Additionally, each participant was provided with a detailed explanation of the study, its objectives, and the methodologies we would use. For data collection, we used various digital tools, such as live meetings via Zoom and surveys through Google Forms, ensuring the privacy of the responses at all times.

All participants in this study provided their informed consent before their participation. A written informed consent form was provided, detailing the study objectives, the procedures to be followed, the benefits and potential risks, as well as the confidentiality conditions. Participants signed the document before completing the questionnaire. The study was conducted in accordance with the Institutional Research Ethics Committee of César Vallejo University, under approval number RCU No. 0659-2024-UCV, ensuring compliance with the ethical principles established in the Declaration of Helsinki and the applicable national regulations.

No exemption from consent was granted by the ethics committee, as the study’s methodology involved data collection through a questionnaire. It was considered essential to ensure that each participant explicitly and formally provided their consent.

Additionally, participants were guaranteed the right to withdraw at any time without repercussions, and the confidentiality of the provided information was ensured by using coded responses to prevent any possibility of personal identification.

## Data Availability

Zenodo. Reinventing Conventional Participation: A Model to Mitigate Political Disaffection in Lambayeque, Peru: https://doi.org/10.5281/zenodo.14900621( Villanueva et al., 2025). This article contains the following underlying data: Figure 1. Political_Disaffection.png Note: Created based on questionnaire data, Page 8 of 15
•

data_base_506.xlsx
•
Data_F1000 (1).sav
•
RCU No. 0659-2024-UCV CODE OF ETHICS IN RESEARCH OF THE UCV V02 (2) (1).pdf This project includes the following expanded data: Political_Disaffection.png INFORMED CONSENT (1).pdf Research_Instrument_1000.pdf Data are available under the terms of the Creative Commons Attribution 4.0 International license (CC-BY 4.0). Zenodo. Reinventing Conventional Participation: A Model to Mitigate Political Disaffection in Lambayeque, Peru. https://doi.org/10.5281/zenodo.14900621 ( Villanueva et al., 2025). Data are available under the terms of the Creative Commons Attribution 4.0 International license (CC-BY 4.0).
