# Supplementary material for: Reinventing conventional participation: A model to mitigate political disaffection in Lambayeque, Peru
Source: F1000Res. 2025 Mar 17;14:302. [Version 1] doi: 10.12688/f1000research.162192.1 (PMC13555020; doi:10.12688/f1000research.162192.1)
Supplement: Peer review history

# Reviewer report: Reviewer response for version 1

**DOI:** 10.5256/f1000research.178345.r391007

**Published:** 2025-07-24

**Authors:** Teresa Gil López

**Affiliations:** 1 Department of Social Sciences | Instituto Juan Linz, Universidad Carlos III de Madrid, Getafe, Community of Madrid, Spain

Thank you for allowing me to review the article "Reinventing conventional participation: A model to mitigate political disaffection in Lambayeque, Peru. The study represents loable efforts to improve citizen participation in times of generalized disaffection and distrust of the institutions. The study aimed to **propose a model of conventional participation to mitigate political disaffection** in Lambayeque, Peru. The specific objective was to **determine the factors that lead to political disengagement** in Lambayeque, Peru. Some important contributions arise from this study, however, there are important shortcomings that prevent me from accepting the manuscript from indexing in its current form. I hope that the suggestions that follow can be helpful towards a revised version.

 In general, I think the writing could be improved. For instance, the theoretical framework should start with an introductory sentence or clause before any study is cited, so that the reader know what they are about to delve into, what part of the literature, dealing with wich concepts, etc. The structure of the theoretical framework could also be generally improved. The current text reads very schematic, as if each study was reviewed one by one. They are not integrated into a coherent argument. There are also some expressions left in Spanish which should be translated into English in accordance with the rest of the text.

 Central study constructs should be more explicitly defined. The authors do a good job in defining political disaffection but other concepts are not so clearly explained. Concrete participation models should also be identified in the literature so that the new factors proposed by the current study can be given the necessary emphasis and their novel contributions can be highlighted above and beyond what traditional models of participation offer. There is in principle nothing new with adding variables related to digital technologies to traditional models of conventional participation.

 Generally, there are expressions in the text which require better contextualization: “ Regarding the theoretical foundations of the conventional participation program, it aligns with the contribution…” what is “it”? And which theoretical foundations of which conventional participation model are the authors referring to, concretely? This goes back to explicitly referring to a set of models and clearly specify the concepts that are central to the main theoretical arguments of this study.

 The sampling procedure should be clearly explained. In the introduction, the authors mention that the study used a “randomly selected sample,” but the characteristics of the sample do not resemble those that would have been obtained via randomized selection. Regardless, how the sample was obtained/accessed to and contacted is a necessary piece of information to assess the study’s method. Some of this is explained in the section about consent but information on how those 506 participants where first chosen from a population/participant pool or panel is unclear. Also the instruments for data collection, primarily the scales used to measure participation and political disaffection: are those scales newly created or are they pre-existing scales? What items are they composed of?

 Finally, the technical proposal presented by the study seems disconnected to the study results, and just generally, it is absent from the article until the point it gets introduced. I would recommend the authors to give this proposal greater centrality from the beginning of the article, and even focus the literature review on propositional models rather than solely on a general discussion on conventional participation and disaffection.

 In all, the study’s objective was to determine the factors that lead to political disengagement in Lambayeque, Peru. However, the study fails to reflect what those factors are and the connections that they have to disengagement are not tested either. I would therefore recommend to reconsider the study’s objectives and propose ones that are more realistic given the study’s empirical design and limited scope.

Is the work clearly and accurately presented and does it cite the current literature?

Partly

If applicable, is the statistical analysis and its interpretation appropriate?

Partly

Are all the source data underlying the results available to ensure full reproducibility?

Partly

Is the study design appropriate and is the work technically sound?

No

Are the conclusions drawn adequately supported by the results?

No

Are sufficient details of methods and analysis provided to allow replication by others?

Partly

Reviewer Expertise:

Communication and media effects, political participation, digital media, communication processes and mechanisms.

I confirm that I have read this submission and believe that I have an appropriate level of expertise to state that I do not consider it to be of an acceptable scientific standard, for reasons outlined above.

# Response

**Published:** 2025-11-23

**Authors:** IDA BLANCA PACHECO GONZALES

**Affiliations:** DIRECCION DE INVESTIGACION, Universidad Cesar Vallejo, Trujillo, La Libertad, Peru

**Subject: Submission of Revised Manuscript and Response to Reviewer Comments**

 Dear Review

 I hope this message finds you well. I am writing to inform you that I have thoroughly reviewed all the observations made regarding the manuscript *“Reinventing Conventional Participation: A Model to Mitigate Political Disaffection in Lambayeque, Peru”* and have fully addressed each of them.

 In the revised version, the writing has been strengthened, the theoretical framework has been reorganized into a more coherent and integrated structure, and the central concepts of the study have been defined with greater clarity. Additionally, the explanation of the sampling procedure has been corrected, specifying how access to the participant panel was obtained and detailing the composition and origin of the instruments used. Greater prominence has also been given to the technical proposal from the beginning of the article, clearly emphasizing that the main objective of the study was to propose a model of conventional participation to mitigate political disaffection, integrating its theoretical and empirical foundations in alignment with propositional models from the literature.

 Thank you for your valuable comments, which have significantly contributed to improving the quality of the manuscript. I remain at your disposal for any further observations.
